# Preliminary results of the validation study of the italian version of the nature relatedness scale 6 items

**DOI:** 10.1192/j.eurpsy.2021.1363

**Published:** 2021-08-13

**Authors:** G. Rogier, S. Beomonte Zobel, R. Tambelli

**Affiliations:** Dynamical And Clinical Psychology, Sapienza Università di Roma, Rome, Italy

**Keywords:** Savoring, validation, factorial structure, Nature Relatedness

## Abstract

**Introduction:**

The construct of nature relatedness (NR) has received a growing attention in the last decades. Past research suggest that NR may be involved in both positive (e.g. well-being) and negative (e.g. technological addictions) psychological outcome.

**Objectives:**

In addition, some evidences suggest that the construct may be tightly related to emotion regulation capacities. Despite Nisbet et al. (2009) recently developed a short measure to investigate the construct, this has not been still validated in the Italian context.

**Methods:**

We performed three studies to validate the Italian version of the NRS-6 and to extend the nomological network of the construct. In the first study, we tested, throughout Structural Equation Modelling, the factorial structure of the instrument and gender invariance. In the second study, construct validity of the instrument was tested examining correlation pattern between NRS-6 scores and scores obtained on the Connection to Nature Scale, the Difficulties in Emotion Regulation Scale and the Ways of Savoring Checklist. Finally, a longitudinal study tested the temporal stability of the measure and the predictive role of NR on technological addiction.

**Results:**

Data documented a good factorial structure of the instrument, satisfying invariance proprieties and a good test-retest reliability. Also, analyses supported the good convergent and predictive validity of the instrument.

**Conclusions:**

The Italian version of the NRS-6 appears a reliable and useful tool that can be used in future research. Our studies extend the nomological network of the construct shedding light on the tight relationship of NR with the capacity to regulate positive emotions.

